# FOXC1 expression and radiological predictors of peritumoral brain edema in meningiomas

**DOI:** 10.1007/s11060-026-05441-6

**Published:** 2026-02-03

**Authors:** Alim Emre Basaran, Max Braune, Alonso Barrantes-Freer, Wolf C. Mueller, Martin Vychopen, Erdem Güresir, Johannes Wach

**Affiliations:** 1 Department of Neurosurgery, University Hospital Leipzig, University of Leipzig, 04103 Leipzig, Germany; 2 Comprehensive Cancer Center Central Germany, Partner Site Leipzig, 04103 Leipzig, Germany; 3 Paul-Flechsig-Institute of Neuropathology, University Hospital Leipzig, 04103 Leipzig, Germany

**Keywords:** Meningioma, FOXC1, Peritumoral brain edema, Immunohistochemistry, Radiomics, WHO grade, Biomarkers

## Abstract

**Background:**

Peritumoral brain edema (PTBE) is a frequent finding in meningiomas and can significantly affect perioperative outcomes. In addition to well-established clinical, radiological, and histopathological risk factors, molecular markers such as Forkhead Box C1 (FOXC1) may also play a crucial role in the development of PTBE.

**Methods:**

We conducted a retrospective, single-center study including 86 patients with histopathologically confirmed meningiomas. Preoperative MRI datasets were analyzed for tumor characteristics using 3D Slicer and ImageJ. FOXC1 expression was assessed immunohistochemically and dichotomized based on receiver operating characteristic (ROC) curve analysis. Univariate analyses were performed to evaluate associations between clinical, radiological, and histopathological variables and the presence of PTBE.

**Results:**

PTBE was significantly associated with low FOXC1 expression (*p* = 0.015). Furthermore, WHO grade 2/3 meningiomas (*p* = 0.012), perioperative seizures (*p* = 0.024), subtype (*p* = 0.016), tumor laterality (*p* = 0.04), higher MIB-1 index *(p* < 0.001) and tumor volume (*p* = 0.01) were also significantly associated with PTBE. Tumor localization (skull base vs. non-skull-base) and sex showed no significant correlation.

**Conclusion:**

Combining molecular and radiological parameters could improve neurosurgical planning and perioperative management. Further studies are needed regarding the assessment of the response to anti-edematous therapies in low and high FOXC1 expressing meningiomas.

**Supplementary Information:**

The online version contains supplementary material available at 10.1007/s11060-026-05441-6.

## Introduction

Peritumoral brain edema (PTBE) is a common finding in meningiomas and can significantly affect clinical management as well as perioperative outcomes. PTBE is present in up to 67% of meningiomas and is associated with increased intracranial pressure, complex perioperative surgical management, higher risk of seizures, postoperative intracranial hemorrhage, tumor recurrence, and increased mortality rates [1–4]. The pathophysiology of PTBE is multifactorial and remains incompletely understood. In addition to mechanical factors such as impaired venous drainage due to compression of cerebral veins and direct compression of adjacent brain parenchyma by the tumor mass, the release of vascular endothelial growth factor (VEGF) and matrix metalloproteinase-9 (MMP-9) has been reported to stimulate vascular permeability, thereby contributing to PTBE formation [5–8]. Previous studies have identified several clinical, radiological, and histopathological parameters as risk factors for PTBE. These include feret diameter, tumor surface area, non–skull-base localization, higher WHO grade, elevated MIB-1 index, and specific histological subtypes [9–15]. However, these factors alone cannot fully explain the occurrence of PTBE, suggesting that additional molecular factors and genetic alterations are likely involved.

Forkhead Box C1 (FOXC1) plays a crucial role in the development of meningeal vessels, the maintenance of blood–brain barrier integrity, and the regulation of vascular permeability [16, 17]. In several tumor entities, including gliomas, FOXC1 has been shown to be involved in tumor biology. Alterations in FOXC1 expression have been associated with increased cell proliferation, cancer stem cell maintenance, tumor cell migration, and angiogenesis [18, 19]. 

To our knowledge, no studies have investigated the correlation between FOXC1 expression and the occurrence of PTBE. Therefore, the aim of the present study was to investigate the association between FOXC1 expression, radiological tumor parameters, and the occurrence of PTBE in histopathologically confirmed meningiomas. In addition, we sought to determine whether FOXC1 is associated with PTBE and whether this association differs between benign (WHO grade 1) and atypical/anaplastic (WHO grade 2/3) meningiomas.

## Materials and methods

The present retrospective study included patients diagnosed with meningioma between 2016 and 2024 who were treated at the Department of Neurosurgery, University Medical Center Leipzig. All patients underwent standardized perioperative planning and care. Patient identification was based on medical records, including clinical documentation and radiological imaging. Histopathological diagnosis of meningioma and WHO grading were performed according to the respective valid World Health Organization (WHO) Classification of Tumors of the Central Nervous System at the time of initial diagnosis. Cases diagnosed before 2021 were graded according to the 2016 classification (4th edition), whereas cases diagnosed from 2021 onward were graded according to the 2021 classification No systematic re-evaluation of earlier cases according to the 2021 criteria was performed, as the grading criteria for meningiomas changed only minimally between the two editions (most notably the explicit confirmation of brain invasion as an independent criterion for WHO grade 2, which was already widely applied under the 2016 classification) [20, 21]. All histopathological diagnoses and gradings were confirmed by board-certified neuropathologists at our institution.

### MRI evaluation of tumor image characteristics and PTBE

Tumor location, tumor volume, surface area, sphericity, elongation, and feret diameter were assessed based on preoperative T1-Gadolinium (Gd)-enhanced magnetic resonance imaging (MRI). The analysis was performed using the 3D Slicer software. The workflow for segmentation and quantification of these radiomic tumor features in meningioma has been described previously [22]. The shape-based radiomic features sphericity, elongation, and flatness were calculated from principal component analysis of the tumor volume. Sphericity measures overall roundness relative to a perfect sphere (range 0–1; 1 = perfect sphere). Elongation quantifies the relationship between the two largest principal axes (range 0–1; 1 = spherical, lower values indicate a more rod-like shape). Flatness quantifies the relationship between the largest and smallest principal axes (range 0–1; 1 = spherical, lower values indicate a more disc-like or flattened shape) [23, 24]. The presence or absence of PTBE was evaluated on preoperative MRI using the FLAIR sequence. In addition, tumor and PTBE signal intensities were measured using ImageJ in T1-Gd-enhanced and FLAIR-weighted MRIs, respectively [25, 26]. 

### Immunohistochemical staining of FOXC1

**Fig. 1:**
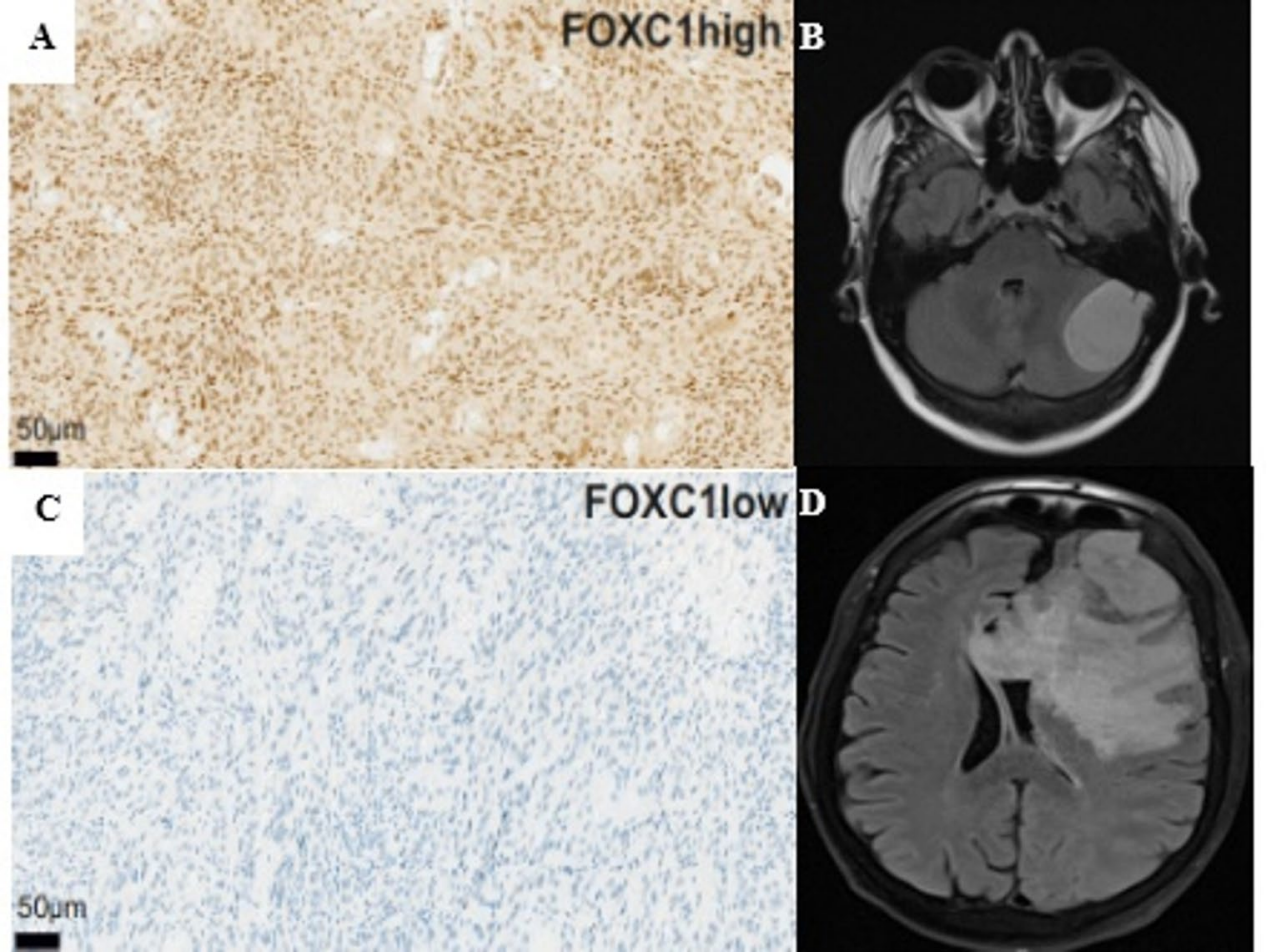
Representative immunohistochemical staining for FOXC1 and corresponding preoperative axial T2-FLAIR MRI sequences in meningioma cases with and without peritumoral brain edema (PTBE). (**A**) High nuclear FOXC1 expression in a WHO grade 1 meningioma without PTBE. (**B**) Corresponding axial T2-FLAIR MRI of the same patient showing no PTBE. (**C**) Low or absent nuclear FOXC1 expression in a WHO grade 3 meningioma with PTBE. (**D**) Corresponding axial T2-FLAIR MRI of the same patient demonstrating extensive PTBE. FOXC1 immunoreactivity was predominantly nuclear, with occasional cytoplasmic staining

Immunohistochemical staining for FOXC1was performed on 1 μm-thick, formalin-fixed, paraffin-embedded (FFPE) patient-specific meningioma tissue sections, following standard protocols of the Paul Flechsig Institute of Neuropathology at the University Hospital Leipzig. Briefly, a primary polyclonal anti-FOXC1 antibody (HPA040670, Sigma; rabbit) was used at a 1:50 dilution, according to the manufacturer’s protocol, on a Ventana BenchMark ULTRA system. (Fig. 1). Whole-slide images of FOXC1-stained sections were scanned for digital quantification of FOXC1-positive tumor cells. Regions of interest were manually selected to ensure that only solid tumor areas were analyzed, while adjacent non-neoplastic tissue such as brain parenchyma or meninges was excluded. Quantification was performed in QuPath (Version 0.5.1) using the positive cell detection function (score compartment: nuclear DAB OD mean) and FOXC1-positive cells were expressed as a ratio of the total number of detected cells [27]. 

### Statistical analysis

All statistical analyses were performed using SPSS version 29 (*IBM*,* Armonk*,* NY*,* USA*). Significant variables were dichotomized based on receiver-operating characteristic (ROC) curve analysis to determine the optimal cut-off. Depending on data distribution, comparisons between groups with and without PTBE were performed using either the independent t-test or the Mann–Whitney U test for continuous variables and the chi-square test or Fisher´s exact test for categorial variables. In addition to univariate analyses, a multivariable binary logistic regression was performed to assess whether FOXC1 expression is an independent predictor of PTBE after adjusting for confounders. Variables included in the model were those significantly associated with PTBE in univariate analysis (*p* < 0.05) or deemed clinically relevant based on prior literature: WHO grad, FOXC1 expression, tumor volume, and MIB-1 index. Odds ratios (OR) with 95% confidence intervals (CI) were calculated for each predictor. A *p*-value < 0.05 was considered statistically significant. Receiver operating characteristic (ROC) curves, violinplot and raincloud plots were generated using the R package ggplot2 (*R Foundation for Statistical Computing*,* Vienna*,* Austria*).

## Results

### Patient characteristics

The present study included 86 patients with cranial meningiomas. The median age at diagnosis was 63 years (IQR: 54–75). Of these, 49 patients (57.0%) were female and 37 (43.0%) were male. Peritumoral brain edema was present in 50 patients (58.1%), whereas 36 patients (41.9%) showed no signs of edema. According to the WHO classification, 61 patients (70.9%) had grade 1, 17 patients (19.8%) had grade 2, and 8 patients (9.3%) had grade 3 meningiomas. Regarding tumor laterality, the lesion was in the right hemisphere in 46 patients (53.5%), in the left hemisphere in 35 patients (40.7%) and showed bilateral hemispheric involvement in 5 patients (5.8%). A skull base localization was observed in 32 patients (37.2%), whereas 54 patients (62.8%) had non–skull-base tumors. Perioperative seizures occurred in 17 patients (19.8%), while 69 patients (80.2%) did not experience seizures. With respect to FOXC1 expression, 44 patients (51.2%) showed high expression levels, whereas 42 patients (48.8%) had low expression. The median MIB-1 index across the cohort was 5% (IQR: 3–8%). For detailed patient characteristics, see Table 1. Baseline characteristics are additionally visualized in the oncoprint heatmap (Supplementary Figure S1), which enables inspection of individual patient profiles and potential associations between variables.

**Table 1:** Patient characteristics

Parameter	Value
**Age at surgery**,** years (median [IQR])**	63 (54–75)
**Sex**	
Male Female	37/ 86 (43.0%) 49/ 86 (57.0%)
**Edema**	
Present Not present	50/ 86 (58.1%) 36/ 86 (41.9%)
**WHO Grade**	
1 2 3	61/ 86 (70.9%) 17/ 86 (19.8%) 8/ 86 (9.3%)
**Laterality of tumor**	
Right Left Both	46/ 86 (53.5%) 35/ 86 (40.7%) 5/ 86 (5.8%)
**Tumor location**	
Skull base Non skull base	32/ 86 (37.2%) 54/ 86 (62.8%)
**Perioperative seizures**	
Present Not present	17/ 86 (19.8%) 69/ 86 (80.2%)
**FOXC1**	
High low	44/ 86 (51.2%) 42/ 86 (48.8%)
**MIB-1 (median [IQR])**	5 (IQR: 3–8)

### Relationship between radiological parameters and PTBE

Tumors with PTBE were associated with larger feret diameters (*p* = 0.006), tumor volume (*p* = 0.01) and larger surface areas (*p* < 0.001) compared to tumors without PTBE. In contrast, other radiological features showed no significant association with PTBE. Tumor intensity (*p* = 0.081) demonstrated a nonsignificant trend toward higher values in patients without edema, while sphericity (*p* = 0.643), flatness (*p* = 0.937), and elongation (*p* = 0.189) did not differ between tumors with and without PTBE. For a summarized overview see Table 2.

**Table 2:** Radiological tumor characteristics in patients with and without postoperative PTBE

Parameter	No PTBE	PTBE	*p*-value
Tumor intensity	1.94 (IQR: 1.75–2.07)	1.74 (IQR: 1.37–2.01)	0.081
Tumor volume in cm^3^	15.05 (IQR: 5.48–21.55)	29.97 (IQR: 16.69–50.36)	*0.01*
Feret diameter in mm	55.0 (IQR: 48.84–62.04)	69.45 (IQR: 57.27–93.57)	*0.006*
Tumor surface area in cm^2^	55.23 (IQR: 38.84–67.03)	106.10 (IQR: 73.71-164.87)	*0.001*
Sphericity*	0.48 (IQR: 0.39–0.62)	0.44 (IQR: 0.38–0.49)	0.643
Flatness*	1.22 (IQR: 1.16–1.39)	1.25 (IQR: 1.16–1.39)	0.937
Elongation*	1.21 (IQR: 1.10–1.44)	1.19 (IQR: 1.10–1.29)	0.189

* Shape features (sphericity, elongation, flatness) were derived from principal component analysis of the tumor volume as defined by PyRadiomics [24]

### Correlation between PTBE and FOXC1 expression in meningiomas

In our analysis, we examined the relationship between PTBE and FOXC1 expression. FOXC1 expression levels were dichotomized, and the optimal cut-off value was determined using ROC curve analysis (cut-off ≤ 26, AUC = 0.69, 95% CI: 0.56–0.79, yielding a sensitivity of 62% and a specificity of 63.9%, indicating a moderate discriminative ability for the presence of PTBE (Supplementary Fig. 3). The distribution of FOXC1 nuclear positivity in meningiomas by WHO grade is visualized in a violin plot with overlaid boxplots and raw data (Supplementary Fig. 4A). In our cohort, low FOXC1 expression was significantly associated with the presence of PTBE, whereas high FOXC1 expression was more frequently observed in patients without PTBE. This inverse relationship between PTBE and FOXC1 expression was statistically significant (*p* = 0.006).

To evaluate whether the relationship between FOXC1 expression and PTBE differed by tumor grade, we performed a subgroup analysis according to the WHO classification. FOXC1 expression decreased with increasing tumor grade. Grade 1 tumor showed the highest FOXC1 levels (35.68 ± 23.78), followed by grade 2 tumors (25.41 ± 32.7) and grade 3 tumors showed the lowest expression (6.77 ± 8.74). FOXC1 expression differed significantly across WHO grades overall (*p* < 0.001). In pairwise comparisons, FOXC1 expression was significantly lower in grade 3 tumors compared to grade 2 tumors (*p* = 0.03). Although grade 1 tumors tended to have higher FOXC1 expression than grade 2 tumors, this difference did not reach statistical significance (*p* = 0.09). Supplementary Fig. 4A summarizes these findings.

The distribution of FOXC1 expression values in tumors with and without PTBE is visualized in a raincloud plot, showing a left-shifted distribution of FOXC1 expression in tumors with PTBE. FOXC1 expression was higher in patients without PTBE (39.5 ± 24.52) compared to those with PTBE **(**24.81 ± 25.79; *p* = 0.01), as visualized in Supplementary Fig. 4B.

### Further demographic, clinical, and pathological characteristics in relation to PTBE

In addition to radiological parameters, we analyzed clinical and histopathological variables, including patient age, sex, presence of perioperative seizures, tumor laterality, location (skull base vs. non–skull-base), WHO grade, histopathological subtype, and MIB-1 index, to assess their association with the presence of PTBE. Statistically significant associations with PTBE were identified for FOXC1 expression (p = 0.015), WHO grade (p = 0.012), histopathological subtype (p = 0.016), perioperative seizures (p = 0.024), tumor laterality (p = 0.04), whereas no significant associations were observed for sex (p = 0.511) and tumor localization (skull base vs. non–skull-base) (p = 0.239). Notably, tumors with PTBE exhibited a significantly higher mean MIB-1 index compared with those without PTBE (3 ± 2.96 vs. 5 ± 6.17, p < 0.001), indicating a correlation between higher proliferative activity and edema formation. For a summarized overview see Table 3. No adjustment for multiple comparisons was applied in these exploratory univariate analyses. To further assess potential confounding by tumor aggressiveness, the association between dichotomized FOXC1 expression and PTBE was examined separately in WHO grade 1 and WHO grades 2/3 meningiomas using Fisher’s exact test. In WHO grade 1 tumors (n = 61), low FOXC1 expression was associated with PTBE in 59.6% of cases compared to 37.5% in high FOXC1 expression, but this difference was not statistically significant (p = 0.353). In WHO grades 2/3 tumors (n = 25, a substantially smaller subgroup), the prevalence of PTBE was 76.9% in low FOXC1 expression and 83.3% in high FOXC1 expression (p = 0.659). The limited number of higher-grade cases reduces statistical power in this subgroup. These stratified analyses suggest that the significant univariate association observed in the overall cohort is not consistently present within grade subgroups and is likely influenced by the correlation between FOXC1 expression and tumor grade.

**Table 3:** Comparison of molecular, histopathological and clinical characteristics between patients with and without postoperative PTBE

Parameter	No PTBE	PTBE	*p*-value
**FOXC1 expression**			
Low High	12/ 36 (33.3%) 24/ 36 (66.7%)	30/ 50 (60.0%) 20/ 50 (40.0%)	*0.015*
**WHO grade**			
1 2 3	31/ 36 (86.1%) 5/ 36 (13.9%) 0/ 36 (0%)	30/ 50 (60.0%) 12/ 50 (24.0%) 8/ 50 (16.0%)	*0.012*
**Subtype**			
Angiomatous Meningothelial Microcystic Fibrous Lymphoplasmacytic Secretory Atypical Anaplastic Transitional	2/ 35 (5.7%) 11/ 35 (31.4%) 7/ 35 (20.0%) 3/ 35 (8.6%) 0/ 35 (0.0%) 0/ 35 (0.0%) 5/ 35 (14.3) 0/ 35 (0.0%) 7/ 35 (20.0%)	1/ 49 (2.0%) 15/ 49 (30.6%) 2/ 49 (4.1%) 0/ 49 (0.0%) 1/ 49 (2.0%) 2/ 49 (4.1%) 12/ 49 (24.5%) 8/ 49 (16.3%) 8/ 49 (16.3%)	*0.016*
**Perioperative seizure**			
Not present Present	33/ 36 (91.7%) 3/ 36 (8.3%)	36/ 50 (72.0%) 14/ 50 (28.0%)	*0.024*
**Sex**			
Male Female	14/ 36 (38.9%) 22/ 36 (61.1%)	23/ 50 (46.0%) 27/ 50 (54.0%)	0.511
**Laterality**			
Left Right both	9/ 36 (25.0%) 24/ 36 (66.7%) 3/ 36 (8.3%)	26/ 50 (52.0%) 22/ 50 (44.0%) 2/ 50 (4.0%)	*0.04*
**Localization**			
Skull base Non skull base	16/ 36 (44.4%) 20/ 36 (55.6%)	16/ 50 (32.0%) 34/ 50 (68.0%)	*0.239*
**MIB-1 index**	3 ± 2.96	5 ± 6.17	*< 0.001*

### Multivariate analysis of FOXC1, clinical, and radiological parameters associated with PTBE

To determine independent predictors of PTBE, a binary logistic regression model was constructed including WHO grade, FOXC1, tumor volume, MIB-1 index, and tumor laterality (left-sided vs. right-sided as reference category; bilateral cases excluded due to low number). In the multivariable logistic regression model, tumor volume emerged as the only independent factor significantly associated with the outcome (OR = 0.094, 95% CI 0.019–0.466, *p* = 0.004), indicating a strong inverse relationship.

**Fig. 2:**
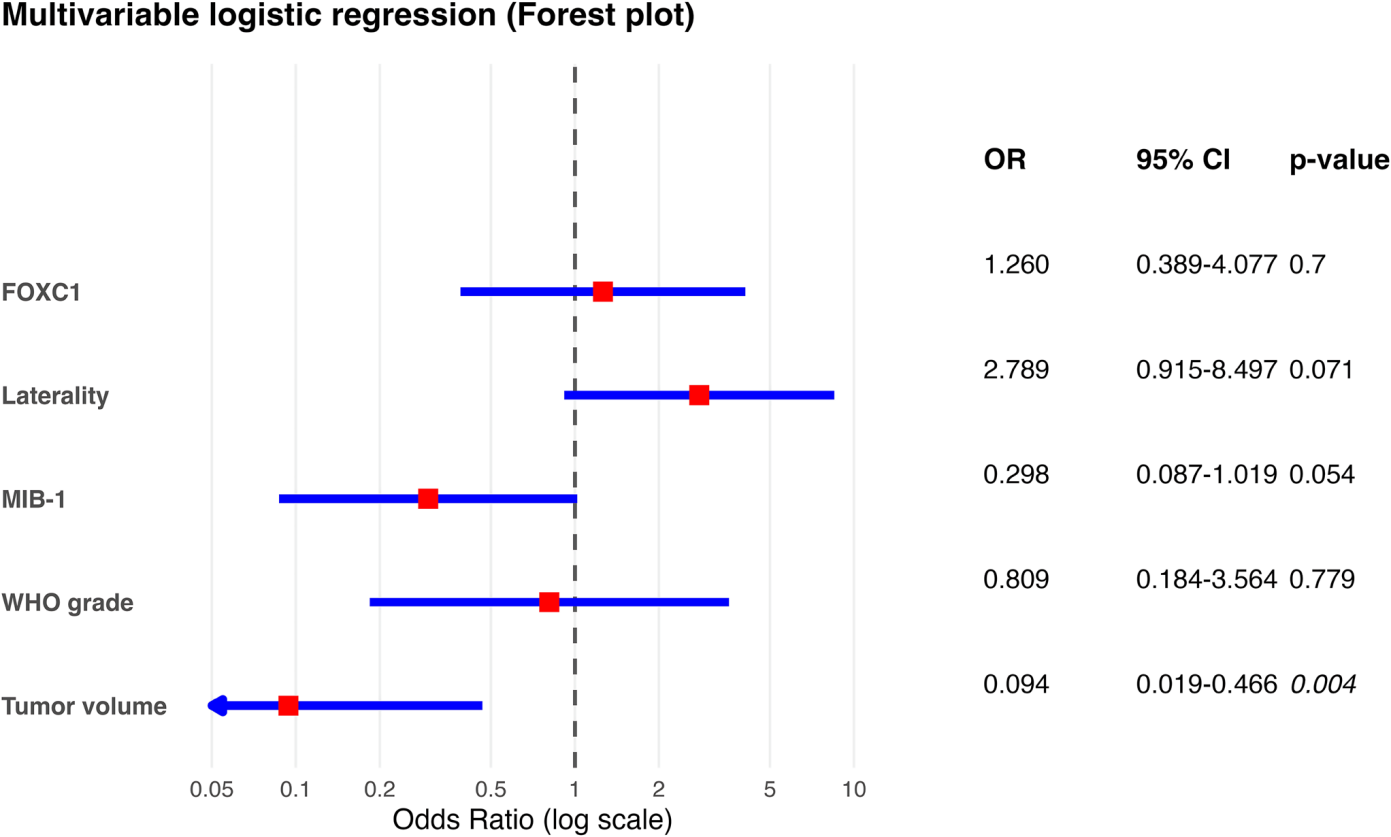
Forest plot of the multivariable logistic regression analysis showing predictors of PTBE. Odds ratios (ORs) with corresponding 95% confidence intervals (CIs) are displayed on a logarithmic scale. Red squares indicate point estimates of the ORs, while blue horizontal lines represent 95% CIs. The vertical dashed line denotes the line of no effect (OR = 1). Confidence intervals extending beyond the plotting range are truncated and indicated by arrows. P-values are shown on the right. Tumor volume was significantly associated with PTBE, whereas FOXC1 expression, laterality, MIB-1 index, and WHO grade did not reach statistical significance in the multivariable model

In contrast, FOXC1 expression showed no independent association in the multivariable model (OR = 1.260, 95% CI 0.389–4.077, *p* = 0.700). Similarly, WHO grade (OR = 0.809, 95% CI 0.184–3.564, *p* = 0.779) and MIB-1 labeling index demonstrated no statistically significant association, although MIB-1 showed a trend toward lower odds (OR = 0.298, 95% CI 0.087–1.019, *p* = 0.054). Tumor laterality likewise did not reach statistical significance (OR = 2.789, 95% CI 0.915–8.497, *p* = 0.071). Overall, these findings suggest that the associations observed in univariable analyses for FOXC1 expression and tumor laterality were not independent, but rather influenced by interrelated factors, with tumor volume representing the dominant independent predictor in the multivariable setting. A corresponding forest plot illustrating the multivariate odds ratios is provided as Fig. 2.

## Discussion

The aim of the present study was to investigate the association between FOXC1 expression, radiological tumor characteristics, clinical parameters, histopathological with the occurrence of peritumoral brain edema (PTBE) in patients with histopathologically confirmed meningiomas. In univariate analyses, low FOXC1 expression was significantly associated with the presence of PTBE. Furthermore, larger feret diameter, increased tumor volume, greater surface area, higher WHO grade (WHO grade 2/3), histopathological subtype, perioperative seizures, tumor laterality, and higher MIB-1 index were all significantly associated with PTBE. In contrast, including sex and tumor localization (skull base vs. non skull base) showed no significant association with the presence of PTBE.

Importantly, when these variables were analyzed simultaneously in a multivariable logistic regression model, tumor volume emerged as the only independent predictor of PTBE. In contrast, the associations observed for FOXC1 expression, WHO grade, MIB-1 index, and tumor laterality in univariate analyses were attenuated and no longer reached statistical significance. These finding highlights tumor volume as the dominant determinant of PTBE formation, likely reflecting its integrative role in capturing cumulative biological effects such as tumor–brain interface disruption, angiogenesis, and vascular permeability.

To our knowledge, this is the first study to demonstrate a significant correlation between FOXC1 expression and the presence of PTBE in meningiomas. FOXC1 is a member of the Forkhead box family of transcription factors and has been associated with tumor progression, epithelial–mesenchymal transition, and the regulation of angiogenesis [28, 29]. In our cohort, low FOXC1 expression was significantly associated with the occurrence of PTBE (71.4% in FOXC1 low vs. 45.5% in FOXC1 high). FOXC1 is a crucial transcriptional regulator of vascular integrity, playing an essential role in the development of meningeal vessels and the maintenance of the blood–brain barrier. Mutations or reduced expression of FOXC1 have been linked to abnormalities in meningeal vessels, structural defects in vessel walls, and consequently increased vascular permeability, which promotes PTBE formation. Preclinical models have shown that reduced FOXC1 expression impairs the formation of the basement membrane, further contributing to increased vascular permeability. Genome-wide analyses in patients with small vessel disease have identified FOXC1 mutations, and MRI findings in these patients demonstrated extensive brain edema and impaired vascular integrity [30–32]. FOXC1 has also been implicated in inflammatory processes: experimental studies revealed that FOXC1 overexpression reduces neuroinflammation and microglial migration, thereby potentially limiting edema formation [33]. This supports a protective role of FOXC1 against inflammation-related edema development. In meningioma research, FOXC1 has further been described as a marker of tumor differentiation and immune competence. Limited studies have suggested that high FOXC1 expression is associated with a preserved vascular and immunological microenvironment [34, 35]. 

Our findings regarding PTBE and its association with atypical/anaplastic meningiomas (WHO grades 2 and 3) showed a statistically significant correlation, indicating that the occurrence of PTBE increases with WHO grade. Compared with WHO grade 1 tumors, grade 2 and 3 meningiomas exhibited a markedly higher prevalence of PTBE. These results are consistent with current literature. In a retrospective study of 240 patients, Ressel et al. [13] reported a significant association between PTBE and WHO grade, with PTBE being significantly more extensive in grade 2 and 3 tumors than in grade 1 meningiomas. Similarly, Reszec et al. [36] demonstrated that atypical/anaplastic meningiomas (based on WHO 2007 classification, grades 2 and 3) exhibit an increased number of mast cells and elevated hypoxia-inducible factor-1 (HIF-1) activity, both of which were significantly associated with the extent of PTBE. These findings underline the link between higher WHO grade, hypoxia, and mast cell activity, all of which appear to play a key role in PTBE formation. Further studies have also confirmed the association between increasing WHO grade and the presence of PTBE [10, 37–39]. 

In the present study, we found that tumor laterality was significantly associated with PTBE. Left-sided tumors showed the highest prevalence of PTBE (74.3%), whereas right-sided (47.8%) and bilateral tumors (40%) exhibited substantially lower rates. To our knowledge, no previous study has specifically investigated the relationship between hemispheric tumor laterality and the presence of PTBE. Most prior studies focused on tumor diameter, WHO grade and anatomical location (convexity, parasagittal and skull base vs. non-skull base) [40]. Laajava et al. [38] demonstrated that PTBE frequently persists even after gross-total tumor resection. However, tumor laterality was only reported only descriptively and not evaluated as a potential contributing factor. Toh et al. [41] showed that impaired glymphatic function is closely associated with the extent of PTBE. Although hemispheric differences were not examined in their study, their findings suggest that neuroanatomical variations in perivascular clearance mechanisms may influence edema formation. In this context, it is plausible that structural or functional hemispheric differences in glymphatic or venous drainage pathways could contribute to the higher prevalence of PTBE observed in left-sided tumors in our cohort. However, tumor laterality was relatively balanced in our cohort (right-sided: 53.5%, left-sided: 40.7%, bilateral: 5.8%), and no major imbalances in confounding factors such as tumor volume, WHO grade, or histological subtype were observed between hemispheres. Given the absence of consistent reports of lateralization effects on PTBE in the literature, this finding should be interpreted cautiously and warrants validation in larger, independent cohorts. Furthermore, when tumor laterality was included in multivariate logistic regression adjusting for WHO grade, FOXC1 expression, tumor volume, and MIB-1 index, the association did not remain statistically significant *(p* = 0.071), suggesting that the observed effect is likely confounded by other tumor characteristics.

Several studies have shown significant differences between meningioma subtypes and the presence of PTBE edema. In contrast to our cohort, in which meningothelial and atypical subtypes were mostly frequently associated with PTBE, the current literature reports that angiomatous and microcystic meningiomas exhibit the highest rates of PTBE [42, 43]. Furthermore, Osawa et al. [40] in a study including 110 patients, demonstrated that secretory, microcystic and angiomatous subtypes are most strongly linked to extensive PTBE. These discrepancies highlight that the contribution of histological subtype to PTBE formation is likely heterogeneous and may vary between different patient populations, suggesting that additional biological or molecular factors influence edema development beyond histology alone.

Our findings demonstrated a significant correlation between the presence of PTBE and perioperative seizures, which is consistent with the current literature. In a systematic review and meta-analysis, Tanti et al. [44] showed that the extent of PTBE is one of the most important independent risk factors for perioperative seizures in patient with meningiomas. Additional studies likewise highlight this association between PTBE and tumor-related epileptic events [45–47]. Taken together, these findings support our results and emphasize that PTBE is not merely a radiological sign but a clinically relevant marker for preoperative risk assessment.

Our results also demonstrate a significant correlation between an increased MIB-1 index, a marker of cellular proliferation, and the presence of PTBE in our cohort. Several studies have reported an association between higher MIB-1 index values and the occurrence of PTBE [2, 15, 48]. In a retrospective study of 110 patients, Osawa et al. [40] found a significant correlation between PTBE and a higher MIB-1 index, with a cut-off value of ≥ 4%. These findings from the current literature are consistent with our results.

Among the radiological parameters analyzed, feret diameter and surface area emerged as the most relevant predictors of PTBE. Both were significantly increased in tumors associated with PTBE in our cohort. These findings are consistent with the current literature, which has demonstrated a clear association between feret diameter, tumor surface area and PTBE. In a retrospective study of 205 patients with convexity and parasagittal meningiomas, feret diameter was identified as an independent predictor of PTBE, with a cut-off value of 3 cm for its prediction [9, 10]. Regarding surface area, the available literature is more limited. Both Ito et al. [11] and Cai et al. [49] demonstrated that a larger tumor–brain contact surface is significantly associated with the presence and severity of PTBE in meningiomas. Cai et al. further quantified this relationship, reporting an approximately 17% increased risk of PTBE for each additional cm² of contact area.

In the present study, univariate analysis revealed a significant association between low FOXC1 expression and the presence of peritumoral brain edema (PTBE) (*p* = 0.015). Although low FOXC1 expression was significantly associated with PTBE in the univariate analysis, this relationship weakened after multivariable adjustment. When WHO grade, tumor volume, and the MIB-1 index were included in the logistic regression model, FOXC1 no longer reached statistical significance, yet it remained the variable with the most pronounced trend toward an independent association with PTBE (OR = 0.985, 95% CI: 0.966–1.005). This attenuation suggests that the apparent FOXC1–PTBE relationship is at least partly influenced by confounding from markers of tumor aggressiveness. In particular, higher WHO grade and increased proliferative activity (MIB-1) may contribute to PTBE formation and, at the same time, be linked to lower FOXC1 expression, thereby diminishing the independent effect of FOXC1 in the adjusted model. Taken together, our findings support FOXC1 as a potentially relevant biological correlate of PTBE but imply that its impact is intertwined with established aggressive tumor features rather than acting as a fully independent predictor. In WHO grade 1 tumors (*n* = 61), low FOXC1 expression was associated with PTBE in 59.6% of cases compared to 37.5% in high FOXC1 expression, but the difference was not statistically significant (*p* = 0.353). In the substantially smaller subgroup of WHO grades 2/3 tumors (*n* = 25), PTBE prevalence was similar between low (76.9%) and high (83.3%) FOXC1 expression (*p* = 0.659). The limited number of higher-grade cases reduces statistical power in this subgroup. Nevertheless, the biological plausibility supports the contributory role for FOXC1 in PTBE pathophysiology, although no direct functional or mechanistic data are provided in this observational study. Preclinical evidence indicates that FOXC1 plays a crucial role in vascular integrity, pericyte function, and blood–brain barrier maintenance, and reduced FOXC1 activity has been linked to increased vascular permeability and BBB disruption in other model [16, 17, 32] These findings raise the hypothesis that meningiomas with low FOXC1 expression may exhibit a structurally vulnerable vascular phenotype promoting vasogenic edema; however, dedicated functional studies are needed to establish causality in this context.

From a therapeutic perspective, FOXC1 remains a promising biomarker candidate. Patients with low FOXC1-expressing tumors may particularly benefit from intensified anti-edematous strategies, such as corticosteroids or VEGF-targeted therapies, given the established role of VEGF-dependent mechanisms in vascular permeability and PTBE formation [1, 7, 8]. Additionally, experimental data indicate that FOXC1 plays an important role in neuroinflammatory regulation through inhibition of NF-κB–dependent signaling pathways [33]. Low-FOXC1 meningiomas may therefore display both vascular and immunological dysregulation, potentially justifying more aggressive inflammation-modulating approaches. Given the established role of VEGF in PTBE pathogenesis and the clinical use of bevacizumab in symptomatic cases, co-expression analyses of VEGF and FOXC1 would be valuable to elucidate whether low FOXC1 expression correlates with or is independent of VEGF upregulation [50, 51]. However, these proposed clinical implications for perioperative management and therapeutic stratification remain speculative, as the present study did not include analyses of postoperative outcomes, treatment responses, or clinical events. A further translational aspect lies in the integration of FOXC1 into radiogenomic models. Although FOXC1 expression alone showed only moderate discrimanitve ability of for the presence of PTBE (AUC = 0.69, 95% CI: 0.56–0.79), established radiomic parameters such as tumor surface area and feret diameter have already shown promise in predicting PTBE risk, WHO grade, and proliferative activity in a non-invasive manner [9, 12, 49]. Combining FOXC1 with these radiological factors could serve as a supplementary molecular biomarker to support improved preoperative risk stratification and individualized perioperative management.

There are several limitations to the present study. The retrospective and monocentric design may introduce selection bias and limit the generalizability of the findings. Local surgical experience, differences in radiological assessment, and variability in histological sample preparation workflows could also have influenced the results. Second, the relatively small sample size may have limited statistical power. The determined cut-off value for FOXC1 expression was not validated in an independent cohort. Moreover, other molecular markers and genetic alterations potentially contributing to PTBE in this patient population were not investigated. In particular, vascular endothelial growth factor (VEGF), which is well-established as a key mediator of vascular permeability and PTBE in meningiomas, was not assessed in this cohort. Future studies should investigate the relationship between FOXC1 and VEGF expression to determine potential interactions or complementary roles in edema formation. Although the ROC-derived cut-off provided moderate discriminative ability (AUC = 0.69), the lack of external validation represents an important limitation. To our knowledge, this is the first study to examine FOXC1 expression in relation to peritumoral brain edema in meningiomas, and no established cut-off values or validated thresholds for FOXC1 immunohistochemistry in this context currently exist in the literature. Future larger-scale studies should include independent validation cohorts and sensitivity analyses to confirm the optimal dichotomization strategy and clinical utility of FOXC1 as a biomarker. Moreover, other molecular markers and genetic alterations potentially contributing to PTBE in this patient population were not investigated. FOXC1 expression was assessed solely by immunohistochemistry, quantitative mRNA or protein expression analyses were not performed. Additionally, FOXC1 expression exhibited a continuous distribution, necessitating digital image analysis for accurate quantification. The interobserver reliability of manual or semi-quantitative FOXC1 assessment by pathologists was not evaluated. Given the single-center design and modest sample size, external validation of our findings in a larger, preferably multicenter cohort, along with studies assessing interobserver agreement for FOXC1 immunohistochemistry, is required to confirm the observed associations and evaluate clinical applicability. Furthermore, the modest sample size likely limited the power to detect independent effects in multivariate analysis, particularly regarding potential confounding between FOXC1 expression and tumor aggressiveness (WHO grade).

Additionally, multiple univariate comparisons were performed without adjustment for multiple testing, increasing the risk of type I error and false-positive findings. The reported significant associations should therefore be considered exploratory and hypothesis-generating. Finally, the presence or absence of PTBE was assessed only preoperatively, and postoperative dynamic changes in PTBE were not evaluated.

## Conclusion

In the present study, low FOXC1 expression was significantly associated with the presence of PTBE, particularly in WHO grade 2 and 3 meningiomas. Furthermore, larger feret diameter, tumor surface area, tumor volume, higher WHO grade, tumor laterality, perioperative seizures, histopathological subtype and increased MIB-1 index were significant predictors of PTBE. Our results underscore the need for prospective validation studies to further evaluate these parameters as a potential biomarker for individualized postoperative care in this defined patient subgroup. These findings highlight FOXC1 as a potential biomarker candidate associated with PTBE, especially in grade 2 and 3 meningiomas. However, given the exploratory nature of this single-center study with a modest sample size and automated quantification method, and the absence of outcome-based analyses linking FOXC1 expression to postoperative clinical events or treatment responses, external validation in larger multicenter cohorts, including interobserver agreement studies for FOXC1 immunohistochemistry, is required before clinical implementation. Further studies are needed regarding the assessment of the response to anti-edematous therapies in low and high FOXC1 expressing meningiomas.

## Supplementary Information

Below is the link to the electronic supplementary material.

Supplementary Material 1

## Data Availability

No datasets were generated or analysed during the current study.
