# Supplementary material for: FOXC1 expression and radiological predictors of peritumoral brain edema in meningiomas
Source: J Neurooncol. 2026 Feb 3;176(3):194. doi: 10.1007/s11060-026-05441-6 (PMC12868038; doi:10.1007/s11060-026-05441-6)
Supplement: Supplementary file 1 — Supplementary Material 1 [file 11060_2026_5441_MOESM1_ESM.pdf]

Supplementary Material

Oncoprint of Clinical, Histopathological and Molecular Features in the sporadic cranial meningioma

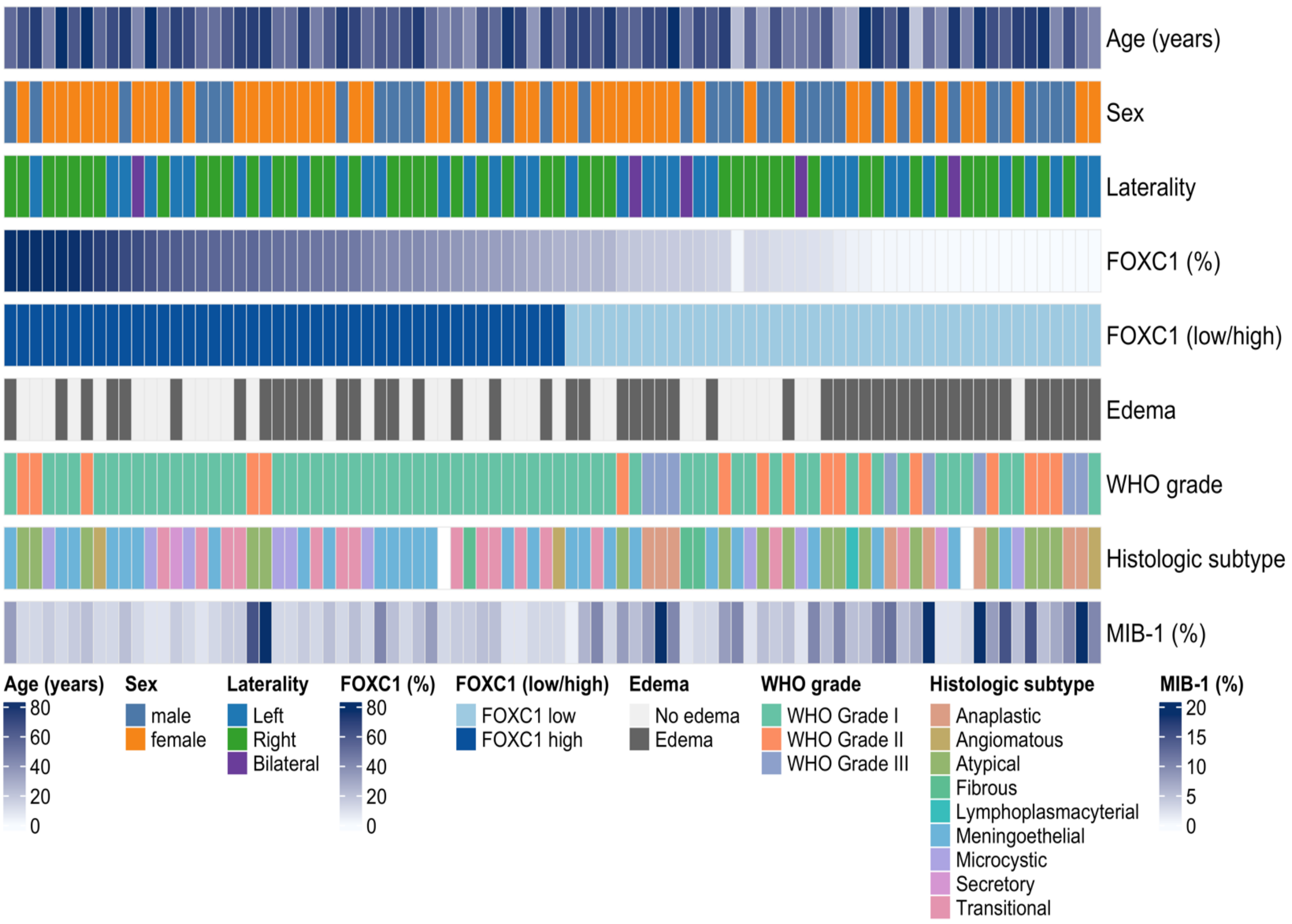

Supplementary Fig. S1: Oncoprint heatmap of clinical, histopathological, and molecular characteristics in the sporadic cranial meningioma cohort (n = 86). Each column represents an individual patient, and each row corresponds to a specific clinical, histopathological, or molecular variable. Categorical variables are represented by distinct color codes: sex (male, 37/86; 43.0%; female, 49/86; 57.0%), laterality (left, 35/86; 40.7%; right, 46/86; 53.5%; bilateral involvement, 5/86; 5.8%), FOXC1 expression category (low, 42/86; 48.8%; high, 44/86; 51.2%), vasogenic edema (absent, 36/86; 41.9%; present, 50/86; 58.1%), WHO grade (grade 1, 61/86; 70.9%; grade 2, 17/86; 19.8%; grade 3, 8/86; 9.3%), and histopathological subtype (WHO grade 2 and 3) [atypical, 17/86; 20.2%; meningothelial, 26/86; 31.0%; microcystic, 9/86; 10.7%; anaplastic, 9/86; 9.5%; angiomatous, 3/86; 3.6%; transitional, 15/86; 17.9%; secretory, 2/86; 2.4%; lymphoplasmacytic, 1/86; 1.2%; fibrous, 3/86; 3.6%]. Summary statistics for each variable are displayed on the right side of the heatmap, including median values with interquartile ranges (IQR) for continuous variables [median age: 63 years (IQR: 54–75), median FOXC1 expression: 26.32% (IQR: 7.52–50.95), median MIB-1 index: 5.0% (IQR: 3.0–8.0)]. This visualization enables a direct comparison of patient-level data and highlights potential patterns or clustering, such as the higher frequency of vasogenic edema in cases with low FOXC1 expression or higher WHO grade.

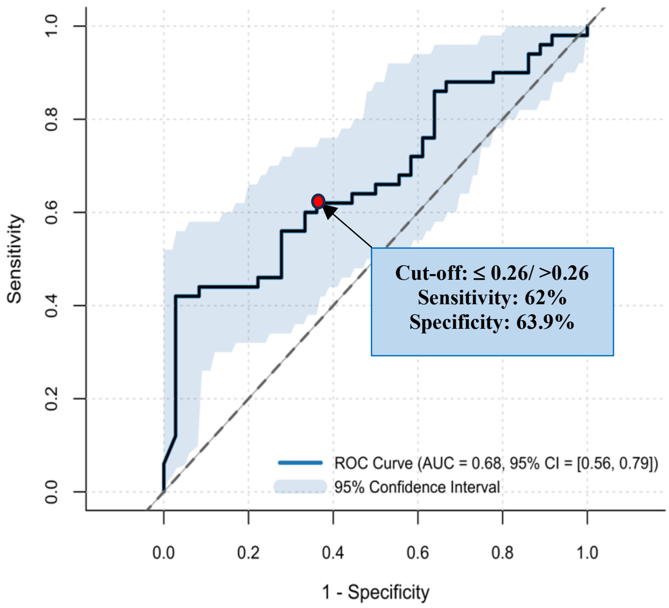

Supplementary Fig. S2: ROC curve analysis for FOXC1 expression to predict the presence of PTBE. The ROC analysis yielded an AUC of 0.69 (95 CI: 0.56-0.79), indicating moderate discriminative ability of FOXC1 expression for PTBE prediction. The optimal cut-off value for FOXC1 expression was ≤0.26, resulting in a sensitivity of 62% and a specificity of 63.9%. The blue line represents the ROC curve, and the shaded area indicates the 95% CI. The red dot marks the optimal cut-off point.

A

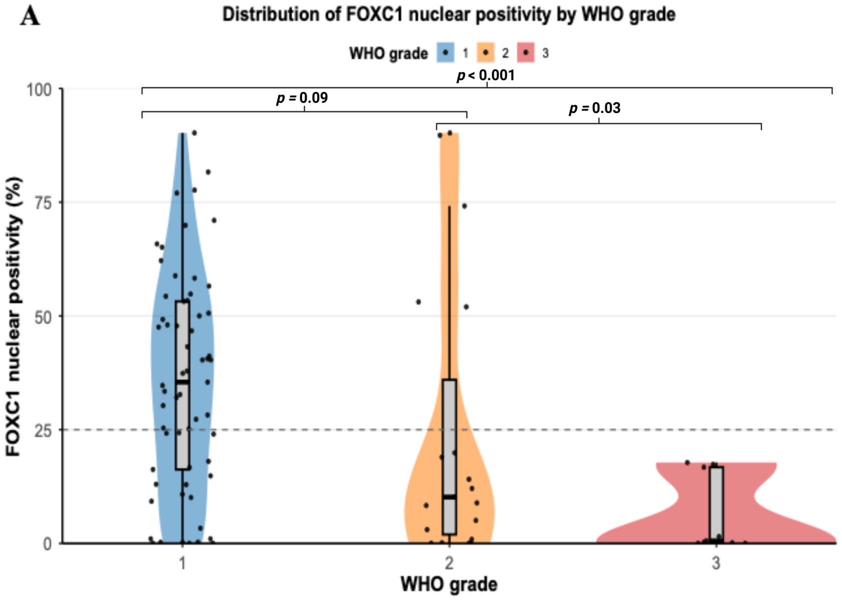

Supplementary Fig. S3A: Distribution of FOXC1 nuclear positivity across WHO grades. Violin plots show the distribution of FOXC1 nuclear positivity (%) in meningiomas stratified by WHO grade (1 = blue, 2 = orange, 3 = red). Thin embedded boxplots indicate the median and interquartile range (IQR). Jittered points represent individual tumors. The dashed horizontal line at 26% marks the cut-off derived from the ROC analysis (see Fig. 3). The y-axis spans 0–100%; higher values indicate greater nuclear FOXC1 immunopositivity on IHC. FOXC1 expression differed significantly between groups (overall $p < 0.001$), with lower expression in WHO grade 2 compared to grade 1 ($p = 0.09$) and in grade 3 compared to grade 2 ($p = 0.03$).

B

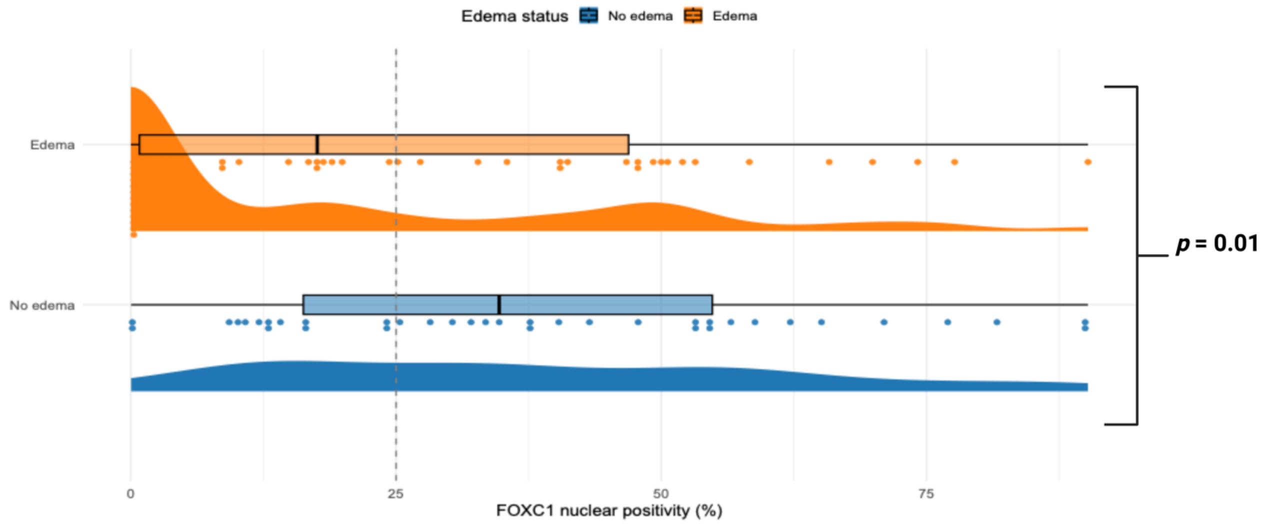

Supplementary Fig. S4B: Raincloud plot illustrating FOXC1 expression levels with and without PTBE. The raincloud plot combines a boxplot, raw data points (dots), and kernel density plots to display the distribution of FOXC1 expression levels between the two groups. Tumor withs PTBE (orange) exhibited lower median FOXC1 expression values and a left-shifted distribution compared to those without PTBE (blue), which showed higher and more widely distributed FOXC1 expression. This visualization highlights the inverse relationship between FOXC1 expression and the presence of PTBE. The dashed vertical line at 26% marks the cut-off derived from the ROC analysis (see **Fig. 3**). FOXC1 expression differed significantly between tumors with and without PTBE ($p = 0.01$).
